# Location Estimation in Wireless Sensor Networks Using Spring-Relaxation Technique

**DOI:** 10.3390/s100505171

**Published:** 2010-05-25

**Authors:** Qing Zhang, Chuan Heng Foh, Boon-Chong Seet, A. C. M. Fong

**Affiliations:** 1 School of Computer Engineering, Nanyang Technological University, 639798, Singapore; E-Mail: ASCHFoh@ntu.edu.sg; 2 Department of Electrical Electronic Engineering, Auckland University of Technology, New Zealand; E-Mail: boon-chong.seet@aut.ac.nz; 3 School of Computing and Mathematical Sciences, Auckland University of Technology, New Zealand; E-Mail: alvis.fong@aut.ac.nz

**Keywords:** wireless sensor networks, localization, cooperative, spring-relaxation technique

## Abstract

Accurate and low-cost autonomous self-localization is a critical requirement of various applications of a large-scale distributed wireless sensor network (WSN). Due to its massive deployment of sensors, explicit measurements based on specialized localization hardware such as the Global Positioning System (GPS) is not practical. In this paper, we propose a low-cost WSN localization solution. Our design uses received signal strength indicators for ranging, light weight distributed algorithms based on the spring-relaxation technique for location computation, and the cooperative approach to achieve certain location estimation accuracy with a low number of nodes with known locations. We provide analysis to show the suitability of the spring-relaxation technique for WSN localization with cooperative approach, and perform simulation experiments to illustrate its accuracy in localization.

## 1. Introduction

Supported by the great development in RF and MEMS IC design, large-scale distributed wireless sensor networks (WSNs) are widely applied to many areas involving monitoring, tracking, and controlling. Specific applications of wireless sensor networks include vehicle surveillance, environment monitoring, health care applications, and home automation [1–5].

Accurate and low-cost autonomous self-localization of the sensors is a critical requirement in these WSNs. The main reason is that the locations of sensors are necessary in a variety of applications, such as environment monitoring, object detection, target tracking, and security surveillance. In these applications, sensor locations must be known to make sense of the reported data. Additionally, sensor location information can be very useful for geographic routing protocols [6], clustering algorithms [7], and geographic data fusion algorithms [8].

Sensor localization is a challenging issue in the design and development of WSNs in various applications [9]. Sensors will need to last for years without battery replacement, so that power consumption and energy efficiency is of great concern. The localization algorithm should be designed in an easily implemented and energy efficient way, so that it will not limit the main operation of the sensors. Moreover, movement of sensors is also a common setup in various applications of WSNs. In these applications, the localization algorithm should be designed to cope with sensor mobility and changing connectivity. In addition, the localization algorithm should be autonomous and self-configuration without significant human attendance.

In general, a localization algorithm is often based on computation using obtained measurements with respect to devices with known absolute locations where the measurements may include ranges or angles in an absolute or a relative description, such as time of arrival (TOA), time difference of arrival (TDOA), angle of arrival (AOA), received signal strength (RSS), and others. The Global Positioning System (GPS) [10] is an example of a localization system that uses TDOA as the measurements for localization. While robust localization systems exist, as the number of nodes of a WSN is usually large, their direct applications to WSNs may result in high cost. The ZiLA algorithm [11] is an example of location estimation algorithm that uses RSS as the measurements for localization. Specifically, it is a Maximum Likelihood Estimator (MLE) under the log-normal models for the RSS measurements for the ZigBee networks.

The attempt to design a practical localization algorithm for WSNs gives rise to the concept of *cooperative* localization [12]. Cooperative localization algorithm is characterized by utilizing the estimated locations of neighboring nodes that are simultaneously performing localization with the same algorithm. In other words, nodes implementing cooperative localization compute locations relative to the locations advertised by their one-hop neighbors. The computed locations are then advertised back to their one-hop neighbors for refinement. This process continues until the computed locations converge. While a cooperative localization algorithm results in relative locations, with inclusion of a small number of devices with known locations, absolute locations of all nodes can be obtained. These devices with known locations, sometimes called beacons or anchors, may make use of manual configuration or other sophisticated localization technique such as GPS to obtain absolute locations. With the design of using devices with known locations to compute locations of others in a cooperative way, the need for devices of known locations are greatly reduced which results in a low-cost localization solution. In addition, cooperative localization algorithm suits well in multi-hop wireless communications which is the common setup in WSNs. Cooperative localization algorithm can be applied to hierarchical WSNs discussed in [13], as well as WSNs discussed in [14].

In this paper, a cooperative localization solution is developed for WSNs. Precisely, a node uses RSS information to estimate distances between itself and other nodes, then it executes algorithm based on spring-relaxation technique. One known spring-relaxation technique used for localization is studied by Priyantha *et al.* in [15] which they call AFL (Anchor-Free Localization). In AFL, sensor nodes start from a random initial coordinate assignment and converge to a consistent solution using only local node interactions. Specifically, AFL is based on two phases. First phase is a fold-free algorithm that coarsely estimates the networks global layout by using a hop-count technique. Second phase optimizes this layout by implementing a mass-spring relaxation, based on more accurate inter-node distances, measured using TOA. AFL is anchor-free and does not require nodes with pre-configured coordinates. It is shown that AFL can produce good coordinate assignments substantially [15]. Meanwhile, AFL also has some side effects [16]. First phase of AFL is centralized, because it can hardly be implemented without a centralized device handling information from nodes. AFL can only be applied to multi-hop networks. In one-hop networks where all nodes are connected each other, AFL fails because the first phase, based on the hop-count, cannot be executed. AFL gives relative location estimates because of its anchor-free nature. Additional effort is required to obtain absolute location estimates. Besides, the location estimation accuracy of AFL is highly dependent on the global-layout generated by first phase. Producing a network with an incorrect layout, without good initial position estimates may cause AFL falsely converge to distorted configurations of the network nodes.

Inspired by AFL, we follow and extend the design concept to develop a fully distributed localization solution specifically for WSNs that are both one-hop and multi-hop. We consider a WSN that consists of a small number of nodes whose locations are known with others whose locations are unknown. Our cooperative localization solution consists of two phases. The first phase alone can be viewed as a non-cooperative localization algorithm whereas the second phase is based on cooperative approach. In the first phase, a sensor node uses RSS to estimate the distances between itself and all visible beacons. These distance inputs are processed using the spring-relaxation technique to determine its location. Due to the small number of beacons and RSS ranging errors [12, 17], this estimated location may give low accuracy. The second phase is designed to refine the estimated location obtained from the first phase. In the second phase, each sensor except beacons exchanges its estimated location information and distance with each of its one-hop neighbors to refine its location again using the spring-relaxation technique. By introducing only a limited number of beacons and applying repeated the spring-relaxation technique in different domains, we aim to provide accurate location estimation with minimal system requirement and deployment effort. Besides, we provide in-depth study on system design and parameter design via both theoretical and experimental approaches. Specifically, we first analyze the convergence property of the spring-relaxation technique in order to ensure that the algorithm terminates within a finite time. We then show that in the presence of RSS ranging noise, cooperative localization with the spring-relaxation technique can ensure stationary outcomes. To further understand the impact of parameters on system performance when concrete wireless ranging characteristics are implemented, we conduct series of simulation experiments to investigate the system behavior by varying the values of design parameters in using the spring-relaxation technique. Finally, we explicitly compare the performance of our proposed solution to other candidates in the same simulation setup.

The rest of the paper is organized as follows. Section 2. presents our localization solution in details, and briefly discusses some design issues. Section 3. provides analysis focusing on the convergence and stationary properties of our solution. Section 4. presents the simulation results covering studies in system design and parameter design, and comparison to other related techniques. Finally, Section 5. summarizes our conclusions.

## 2. Algorithm

We shall now describe our system setup followed by our proposed solution, which is a fully distributed and absolute localization solution specifically designed for both one-hop and multi-hop WSNs. Our considered WSN consists of *N_s_* number of sensors randomly placed onto a map of predefined size with *N_b_* number of beacons. Let 𝕊 and 𝔹 be the sets describing all sensors and beacons respectively, where each sensor is noted as *Sensor_i_*, *i* ∈ 𝕊 and each beacon is noted as *Beacon_j_*, *j* ∈ 𝔹. Each node either a sensor or a beacon is noted as *Node_p_*, *p* ∈ 𝕊 ∪ 𝔹, and vector *V⃗_p_* is used to represent the coordinate of *Node_p_*. Beacons are placed onto the map with fixed coordinates *V⃗_j_*, where *j* ∈ 𝔹. We assume that each beacon is aware of its own absolute location. Whereas each sensor is unaware of its own location, and is configured with an initial guess of location unrelated to its actual deployed location. The two-dimensional (2-D) localization problem is the estimation of *N_s_* unknown-location coordinates *V⃗_i_*, where *i* ∈ 𝕊.

Our algorithm uses RSS as a measure for ranging between a sensor and its one-hop neighbors, which can be either sensors or beacons. Precisely, each *Node_p_* advertises its own location *V⃗_p_* to its one-hop neighbors, where *p* ∈ 𝕊 ∪ 𝔹. Using this advertised transmission, each *Sensor_i_* measures the RSS *s_i,p_* that emitted from the advertising *Node_p_*. According to *s_i,p_*, the distance *d_i,p_* in between is estimated using the path loss model [18]. Using all collected *V⃗_p_* and estimated *d_i,p_*, *Sensor_i_* executes our proposed localization solution based on spring-relaxation technique to achieve accurate estimation of its location. Our proposed localization solution consists of two phases both using the spring-relaxation technique. In the first phase, sensors perform localization based on the location advertisements only from visible beacons, whereas in the second phase, sensors perform localization using the location advertisements transmitted from all neighbors including beacons and sensors. In the following, we first explain the spring-relaxation technique for localization, and then describe the algorithms for the two phases for localization.

### 2.1. Localization Using Spring-Relaxation Technique

To explain the concept of spring-relaxation technique for the localization, we consider a simple example that consists of five beacons and a sensor whose location is to be determined. In the concept of spring-relaxation technique, the considered example is equivalent to having a moving particle (*i.e.*, sensor) attaching with five springs. For each spring, while its one end attaches to the particle, its another end is nailed by a pin (*i.e.*, beacon) at a fixed location.

**Figure 1.**
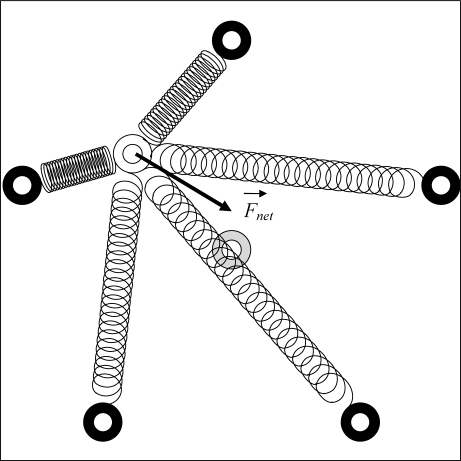
The simple example of five beacons and a sensor.

Figure 1 depicts the described example. In the illustration, the black rings are the beacons or the pins in fixed locations, and white ring is the sensor or the particle in its initial guessed location. The natural length of a spring is the length where the spring is in the rest state. When the length of a spring becomes shorter (resp. longer) than its natural length, the spring is compressed (resp. stretched) and forces are produced at each end of the spring.

The particle attached to a set of springs receives forces from them when they are compressed or stretched. The net force applies on the particle is the vector sum of all received forces. When the particle begins at a particular location with nonzero net force, the net force moves the particle to a new location and the net force changes accordingly. The particle continues to move until the net force becomes zero, and the particle comes to rest. This resting location, indicated by the grey ring in Figure 1, is also the final stopping location of the particle. Localization using spring-relaxation technique does not have real springs connecting the particles and pins. It uses the concept to simulate the movements of the particle under the spring forces computed, and find the final stopping location, which is the estimated location of the particle.

From Hooke’s law, the magnitude of the force *F* from each spring is

(1) $$F=k\left(L_{0}-L\right)$$

where *L*_0_ is the natural length of the spring, *L* is the current length of the spring, and *k* is the spring constant. The difference which *L*_0_ − *L* describes is the stretch or compression of the spring. Let *F⃗* be the force vector applied on a particle by a spring, and $\vec{F_{\mathrm{net}}}=\sum \vec{F}$ be the net force applying on the particle by all the attached springs. By Newton’s first and second law, we have the relationship

(2) $$\vec{F_{\mathrm{net}}}=\frac{d\left(m\;\vec{\upsilon }\right)}{\mathrm{dt}}$$

where *m* is the mass of the particle and *v⃗* is the instantaneous velocity of the particle due to the net force. The instantaneous displacement of the particle can be determined by

(3) $$\vec{D}=\int \vec{\upsilon }\;\mathrm{dt}\;=\iint \frac{\vec{F_{\mathrm{net}}}}{m}{\mathrm{dt}}^{2}.$$

The instantaneous displacement will cause a change in force applied by each spring on the particle. This change in force leads to a new net force on the particle which then changes the instantaneous displacement of the particle again. As this process continues, the particle moves and will eventually rest at an equilibrium location where the net force is zero.

Localization using spring-relaxation technique mimics the behavior of the spring network for the computation of the estimated location. Equations (1)–(2) can be realized similarly in localization. Equation (3) describes that the net force determines the displacement. To ease the computation in (3), the relationship between a force and a displacement can be simplified. AFL uses a simple linear relationship to compute the displacement from a force [15], where in its algorithm, the net force determines the displacement directly. We follow this design principle for our solution.

We will now describe the realization of (1)–(3) in our solution more precisely. Recall the notations defined previously, *V⃗_i_* is the location of *Sensor_i_*, where i ∈ 𝕊; *V⃗_j_* is the location of *Beacon_j_*, where *j* ∈ 𝔹; and *d_i,j_* is the estimated distance between *Sensor_i_* and *Beacon_j_* according to the measured signal strength. Define $\vec{F_{i,j}}$ to be the force that the spring between *Sensor_i_* and *Beacon_j_* exerts on *Sensor_i_*. We show that

$$\vec{F_{i,j}}=\left(d_{i,j}-\left\|{\vec{V}}_{i}-{\vec{V}}_{j}\right\|\right)\times u\left({\vec{V}}_{i}-{\vec{V}}_{j}\right)$$

The scalar quantity (*d_i,j_*− ||*V⃗_i_* – *V⃗_j_*||) is the displacement of the spring from natural length, which gives the magnitude of the force exerted by the spring between *Sensor_i_* and *Beacon_j_*. The unit vector *u*(*V⃗_i_* – *V⃗_j_*) gives the direction of the force on *Sensor_i_*. The spring constant is ignored.

The net force on *Sensor_i_*, defined as *F⃗_i_* is the vector sum of all forces

$${\vec{F}}_{i}=\sum_{j\in 𝔹}\vec{F_{i,j}}$$

To mimic the evolution of the spring network, our algorithm updates the locations of sensors in iterations. In each iteration, the algorithm moves *Sensor_i_* a small distance in the direction of *F⃗_i_* and then recomputes all the applied forces. Let *δ* be the step size of location adjustment. Considering a linear relationship between the net force and the displacement, the location of *Sensor_i_* is then updated as

$${\vec{V}}_{i}\leftarrow {\vec{V}}_{i}+\left(\delta \cdot \vec{F_{i}}\right)$$

### 2.2. Phase 1: Coarse Location Estimation

In this phase, each sensor first makes an initial guess of its location that is irrelevant to its actual location and can be obtained by random assignment, then ranges to each of its visible beacons, and uses the estimated distance from signal strength to iteratively refine this initial guess. Precisely, each *Sensor_i_ i* ∈ 𝕊 first makes the initial guess of *V⃗_i_*, then measures the signal strength from each of its visible *Beacon_j_ j* ∈ 𝔹. Based on the measured signal strength, *s_i,j_*, the distance between *Sensor_i_* and *Beacon_j_* is estimated using the path loss model [18], called *d_i,j_*. *Sensor_i_* also collects the location of *Beacon_j_* which is *V⃗_j_*. After collecting the distances and locations of all its visible beacons, *Sensor_i_* executes the algorithm to estimate its location based on the spring-relaxation technique described previously. Algorithm 1 describes the procedure for *Sensor_i_* to obtain its coarse location estimation.

**Algorithm 1:** Coarse Location Estimation

//For *Sensor_i_*
INPUT: received signal strengths *s_i,j_*, estimated distances *d_i,j_*, beacon locations *V⃗_j_*, and initial guess of *V⃗_i_*
OUTPUT: phase 1 estimate of *V⃗_i_*
*F⃗_i_* = *τ*_1_
**while** ||*F⃗_i_*|| ≥ *τ*_1_**do**
*F⃗_i_* = 0
**for all***Beacon_j_***do**
**if***Beacon_j_* is visible to *Sensor_i_***then**
$\vec{F_{i,j}}=\left(d_{i,j}-\left\|{\vec{V}}_{i}-{\vec{V}}_{j}\right\|\right)\times u\left({\vec{V}}_{i}-{\vec{V}}_{j}\right)$
$\vec{F_{i}}=\;\vec{F_{i}}\;+\;\vec{F_{i,j}}$
**end if**
**end for**
*V⃗_i_* = *V⃗_i_* + *δ*_1_ · *F⃗_i_*
**end while**

In the algorithm, there are several design parameters that are used to adjust the algorithm behavior and control the algorithm execution. Threshold is a constant that used to define the visibility or connectivity. If the received signal strength *s_i,j_* from *Beacon_j_* to *Sensor_i_* is no smaller than the threshold, then *Beacon_j_* is visible to *Sensor_i_*. The specific value of the threshold follows the specification for receiver sensitivity defined in [19]. Tolerance *τ*_1_ is involved in the termination expression of the *while* loop. The iterative update of location estimate in the *while* loop continues executing until the magnitude of the net force ||*F⃗_i_*|| falls below *τ*_1_. Step size *δ*_1_ controls the proportion that *Sensor_i_* updates its location according to the net force in each iteration, that is the convergence speed of the algorithm. Both *τ*_1_ and *δ*_1_ have profound impacts on the algorithm behavior and performance, which is analyzed in later sections.

It should be pointed out that it is not a necessity for every sensor to have visible beacons for practical purpose. Instead, we assume that every sensor has at least three non-collinear neighboring sensors, and this assumption can be easily met in practical applications with large network size and high node density. In multi-hop applications where many sensors reside outside the transmission range of available beacons, those outside sensors will keep their initial guesses as the output of phase 1, and let the refinement process in phase 2 to handle the location estimation with the help of neighboring sensors. The advantage the knowledge of beacon locations constantly brings will be transferred eventually to all sensors in a hop-by-hop manner. As a matter of fact, our localization solution aims to provide accurate location estimation with minimal system requirement and deployment effort, e.g., decentralized structure and low coverage of beacons.

### 2.3. Phase 2: Estimated Location Refinement

After phase 1, a coarse location estimate of all sensor locations is provided. Applying the same spring-relaxation technique in a different domain, phase 2 works on phase 1 estimates to refine the results in a cooperative way. In phase 2, *Sensor_i_ i* ∈ 𝕊 ranges to *Node_p_*, *p* ∈ 𝕊 ∪ 𝔹, *i.e.*, not only visible beacons but also neighboring sensors, to adjust cooperatively their locations according to the net force exerted. It is expected that with the contribution of location information from its neighboring sensors besides that from its visible beacons, a sensor will refine its location estimate in this phase. Meanwhile, similarly to the robot equipped with odometric equipment that moves around to provide appropriate initial coordinates to beacons that discussed in [20], the constant presence of beacons in known locations will impose constrains or rigidity on the available room for force-directed optimizations, and thus avoids the convergence to severe false minima. During the algorithm execution, each sensor will receive position updates from its neighbors, and broadcast its newly updated position to its neighbors. In lightly utilized networks, sensors can manage to use the idle channel to broadcast their position information. In heavily utilized networks, sensors can piggy-back their position information to on-going transmissions. In other words, bandwidth consumption is not a critical issue for the algorithm.

**Algorithm 2:** Estimated Location Refinement

//For *Sensor_i_*
INPUT: received signal strengths *s_i,p_*, estimated distances *d_i,p_*, *V⃗_p_* (including beacon locations *V⃗_j_* and phase 1 estimate of *V⃗_i_*)
OUTPUT: phase 2 refined estimate of *V⃗_i_*
*F⃗_i_* = *τ*_2_
**while** ||*F⃗_i_*|| ≥ *τ*_2_**do**
*F⃗_i_* = 0
**for all***Node_p_***do**
**if***Node_p_* is visible to *Sensor_i_***then**
$\vec{F_{i,p}}=\left(d_{i,p}-\left\|{\vec{V}}_{i}-{\vec{V}}_{p}\right\|\right)\times u\left({\vec{V}}_{i}-{\vec{V}}_{p}\right)$
$\vec{F_{i}}=\;\vec{F_{i}}\;+\;\vec{F_{i,p}}$
**end if**
**end for**
*V⃗_i_* = *V⃗_i_* + *δ*_2_ · *F⃗_i_*
Update the *s_i,p_*, and *d_i,p_*
**end while**

Algorithm 2 describes the procedure for *Sensor_i_* to obtain a more accurate location estimate by refining the coarse location estimate from phase 1. Since phase 2 is also based on the spring-relaxation technique, the algorithm also possesses two design parameters where one describes the tolerance, *τ*_2_ and another describes the step size, *δ*_2_. Due to different consideration in the design, phase 2 shall use a different setting for the parameters. In details, since the second phase follows a cooperative approach, a sensor will gather information not only from visible beacons, but also from other neighboring sensors for the location estimation. With the spring-relaxation technique for localization, the contribution of the net force is much higher for a cooperative approach than that of a non-cooperative approach used in Algorithm 1. Thus, an appropriate setting for Algorithm 2 is where *τ*_2_ > *τ*_1_.

In this phase, the algorithm updates the signal strength and estimation location in every iteration. Due to the noise in the signal strength measures, this constant update causes fluctuation in the computed location. A tighter control in *δ*_2_ is necessary in this phase to maintain the stability of the location estimation. A study on the stability of the location estimation and the influence by *δ*_2_ is given in the following analysis and simulation.

Algorithm 2 can be executed in a continuation manner by excluding the terminate expression of the *while* loop. In this way, the continuous refinement will keep updating the location estimates for applications with either static or dynamic network topology. For static network, continuous refinement can help improving the estimation accuracy, for the effort the infinite RSS sample size makes to eliminate the noise introduced from signal propagation. Whereas for dynamic network, continuous refinement can help adjusting accordingly the estimation to sensor movements.

## 3. Analysis

In this section, we analyze the stability of each phase of the solution. In phase 1, a sensor gathers the location and distance information from each of its visible beacons, and starts the execution of Algorithm 1 using the gathered information. To ensure the algorithm terminates within a finite time indicating its stability, we analyze the convergence of the estimated location.

In phase 2, a sensor exchanges the location and distance information not only from each of its visible beacons, but also from all its neighboring sensors. Moreover, the sensor updates this information constantly during the execution of Algorithm 2. In this cooperative nature of the design, location and distance information fluctuates over the time during the execution, it is thus necessary to test whether the estimated location remain stationary indicating its stability. For this analysis, we show that the estimated location is wide sense stationary.

### 3.1. Convergence Analysis

Our algorithm performs iteration of computation, and in our design, the iteration terminates when the net force falls below a threshold where this weak force has no longer significant contribution to the estimated location of the sensor. Based on extensive simulation experiments, it is indicated in [21] that such an iteration will terminate and that the estimated location will converge to a coordinate. This convergence is also applicable to our algorithm. In the following, we establish the convergence property of our algorithm, where we first investigate this property in a simple one-dimensional setup, and then extend our discussion to the two-dimensional setup.

#### 3.1.1. Convergence in One-Dimensional Setup

We first show the algorithm convergence property in the simple one-dimensional (1-D) setup. We shall use the concept of spring-relaxation for the discussion, which is equivalent to our algorithm. We assume that there is a single location, which we called a *stopping point*, where all forces from beacons cancel out. This assumption is often valid for common configurations.

Consider *n* beacons where *n* ≥ 2. Each beacon is located at *x_i_, i* = 1, 2, …, *n*. All distance variables are normalized to the length of the spring, *i.e.*, the length of the spring is of one unit long. The net force applied on a node located at *x* is

$$F(x)=\sum_{i=1}^{n}F_{x_{i}}(x)$$

where

(4) $$F_{x_{i}}(x)=\{\begin{array}{ll} -x+x_{i}-1, & x<x_{1}\\ 0, & x=x_{i}\\ -x+x_{i}+1, & x>x_{i} \end{array}$$

In the following, we shall prove that in 1-D setup, if there exist a unique stopping point where the net force is zero, at any other location, the net force is nonzero, and the force points towards the stopping point. In (4), we define that the leftward (resp. rightward) force carries a negative (resp. positive) sign.

**Proposition 1** *Consider n beacons where n* ≥ 2. *Each beacon is located at x_i_, i* = 1, 2, …*, n. The net force applied on a node located at x is* $F(x)=\Sigma_{i=1}^{n}F_{x_{i}}(x)$. *If F*(*x*) = 0 *produces a single unique solution, and let the solution be x̂, then* ∀*x* < *x̂, F*(*x*) > 0 *and* ∀*x* > *x̂ F*(*x*) < 0.

**Proof** We first show that ∀*x* < *x̂ F*(*x*) > 0. By (4), *F_x_i__* (*x*) is a monotone decreasing function except at *x* = *x_i_* where the curve steps up by two units. Thus, *F*(*x*) is also a piecewise monotone decreasing function with *n* upward steps occurring at *x_i_*, *i* = 1, 2, …, *n*. In other words, at the discontinuity point *x_i_*, if $F\left(x_{i}^{-}\right)>0$, *F*(*x_i_*) > 0 and $F\left(x_{i}^{+}\right)>0$.

Given that as *x* → −∞, *F*_*x*_*i*__(*x*) → ∞ which implies lim*_x_*_→–∞_ *F*(*x*) > 0. Since *F*(*x*) is a piecewise monotone decreasing function where at any of its discontinuity points *x_i_*, if $F(x_{i}^{-})>0$, then *F*(*x_i_*) > 0 and $F\left(x_{i}^{+}\right)>0$, and *x̂* is the only point that the *F*(*x*) crosses the x-axis, with condition lim*_x_*_→–∞_ *F*(*x*) > 0, in the range where *x* ∈ (−∞, *x̂*), we must have *F*(*x*) > 0.

We now show that ∀*x* > *x̂, F*(*x*) *<* 0, and we shall prove this by using contradiction. Given *F*(*x̂*) = 0 as the unique solution, there exists a point *x* > *x̂* where *F*(*x*) > 0.

Recall that *F*(*x*) is also a piecewise monotone decreasing function with *n* upward steps occurring at *x_i_*, *i* = 1, 2, …, *n* where at any of its discontinuity points *x_i_*, if $F\left(x_{i}^{-}\right)>0$, then *F*(*x_i_*) > 0 and $F\left(x_{i}^{+}\right)>0$. As *x* → ∞, by (4) *F*_*x*_*i*__(*x*) → ∞ which implies lim*_x_*_→∞_ *F*(*x*) *<* 0.

Given that there is a point *x* > *x̂* where *F*(*x*) > 0, since *F*(*x*) is a piecewise monotone decreasing function, and at its discontinuity point *x_i_*, if $F\left(x_{i}^{-}\right)>0$, then *F*(*x_i_*) > 0 and $F\left(x_{i}^{+}\right)>0$, with *F*(*x*) > 0 and lim*_x_*_→∞_ *F*(*x*) *<* 0, there must exist a point *u* ∈ (*x*, ∞) such that *F*(*u*) = 0 which contradict with our condition that *F*(*x̂*) = 0 is the only solution. Thus, if *x* > *x̂* we must have *F*(*x*) *<* 0.

In the above discussion, we make no description on the step size of each movement of the estimated location. The analysis is valid for infinitesimal step size, or equivalently *δ*_1_ is very small. In fact, *δ*_1_ *<* 1 represents the upper bound condition for the setting of *δ*_1_ as *δ*_1_ = 1 corresponds to applying 100% of the net force to the movement of the estimated location.

Moreover, it is possible that a system setup gives more than one stopping points. In practice, such a setup should be avoided as it creates ambiguity in the localization. We here illustrate multiple stopping points using a two-beacon setup. Let *d* be the distance between the two beacons where the two beacons are located at $x_{1}=-\frac{d}{2}$ and $x_{2}=\frac{d}{2}$ respectively. The net force receives by a node at location *x* is *F*(*x*) = *F*_*x*_1__(*x*) + *F*_*x*_2__(*x*). By setting *F*(*x*) = 0, we can solve for locations where the net force is zero. One immediate solution to *F*(*x*) = 0 is *x* = 0. Besides, when *d* < 2, two other solutions can be found, which are *x* = ±1. The condition where *d <* 2 suggests that the two beacons are placed too close to each other. In practical design, beacons should be spread out in location to avoid generating multiple solutions which cause ambiguity in localization.

#### 3.1.2. Convergence in Two-Dimensional Setup

Similar approach can be adopted for the illustration of convergence property in a two-dimensional setup. Consider *n* beacons where *n* ≥ 2. Each beacon is located at coordinate *s_i_*, *i* = 1, 2, …, *n*. All distance variables are normalized to the length of the spring, i.e. the length of the spring is of one unit long. We shall arbitrarily draw a straight line crossing the only stopping point, then rotate the line along with all beacons such that the line becomes horizontal and overlaps with an x-axis, and finally shift the line horizontally such that the stopping point stays at *x̂* on the x-axis. After this transformation, let the xy-coordinate of the *i*-th beacon be (*x_i_*, *y_i_*), *i* = 1, 2, . . ., *n*. We now focus on the image of the forces projected on the straight line, which can be determined by

(5) $$F_{x_{i}}(x)=\left(x-x_{i}\right)\left(\frac{1}{\sqrt{{\left(x-x_{i}\right)}^{2}+y_{i}^{2}}}-1\right)$$

**Figure 2.**
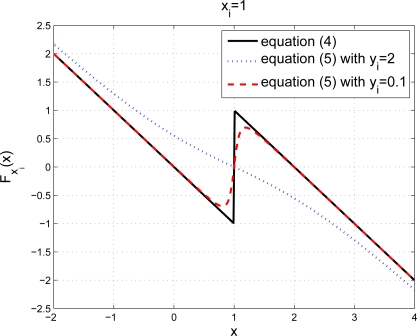
The plot of *F*_*x*_*i*__(*x*) for Equations (4) and (5) with *x_i_* = 1 and different *y_i_* values.

We call the zone where a particular spring experiences compression a compression zone of the spring or the beacon. If the straight line does not cross the compression zone of a beacon, then we have *|y_i_|* > 1 which ensures that the spring remains stretched along the line. It can be shown that with *|y_i_|* > 1, *F*_*x*_*i*__(*x*) given in (5) is a monotone decreasing continuous function. We plot the result of (5) with *x_i_* = 1 and *y_i_* = 2 in Figure 2 to illustrate its monotonic decreasing characteristics.

For the case that the straight line touches or crosses the compression zone of a beacon, we have |*y_i_|* ≤ 1. It can be shown that *F*_*x*_*i*__(*x*) given in (5) decreases monotonically except in the range $(-\sqrt{-y_{i}^{2}+y_{i}^{\frac{4}{3}}}$, $\sqrt{-y_{i}^{2}+y_{i}^{\frac{4}{3}}})$ where it increases monotonically for no more than two units in total. In fact, (4) in the 1-D setup is a special case of (5) where the straight line also crosses the beacon. In Figure 2, the cases of *|y_i_|* ≤ 1 and *y_i_* = 0 both with *x_i_* = 1 are also plotted illustrating a similar characteristics between the case *|y_i_|* ≤ 1 in the 2-D setup and the case *y_i_* = 0 in the 1-D setup.

Using this setup, following the proof given in Proposition 1, it can be shown that with the existence of a unique stopping point, at any other location along the straight line, there exist an net force where its image projected on the straight line points towards the stopping point which moves the node nearer to the stopping point. As the algorithm iterates, the location of the node will eventually converged to the stopping point. Note that since we only show that the image of a force along the line connecting the stopping point and the arbitrary chosen location points towards the the stopping point, the node may not necessarily moves along the straight line back to the only stopping point. The node may move to a new location nearer to the stopping point not necessarily on the straight line, but we can redraw another line for that new location and then apply the same argument to show that the next location of the node will again be nearer to the stopping point than that of the current location.

### 3.2. Stationary Property

**Figure 3.**
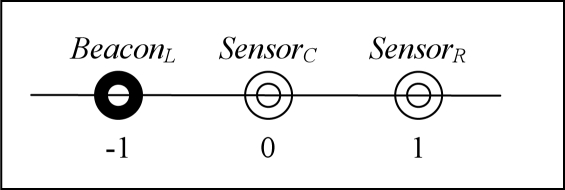
A scenario for a beacon node and two sensor nodes.

For illustration simplicity, we consider a scenario shown in Figure 3. This simple setup consists of a beacon (called *Beacon_L_*) and two sensors (called *Sensor_C_* and *Sensor_R_*). They are placed on the X-axis where *Beacon_L_*, *Sensor_C_*, and *Sensor_R_* are located at −1, 0 and 1 on the X-axis respectively.

We use the notation *C_n_* and *R_n_* to represent the computed sensor coordinates after *n* rounds of iterations in the algorithm. Assume the algorithm is initialized using the true locations, we have *C*_0_ = 0, *R*_0_ = 1. Beacons are always in fixed location, and in this setup *L* = −1. The estimated distance *d* from a particular node to another is calculated according to the path loss model [18] based on the received signal strength. Due to the log-normal shadowing [18] resulting the log-distance path loss model [12, 22], the estimated distance *d* from a node to another is a log-normal random variable of mean *d* and a particular fixed variance *σ*^2^ [22]. Therefore, in the scenario of Figure 3, we have three log-normal random variables for the estimated distances, where *d*_1_ is the estimated distance from *Sensor_C_* to *Sensor_R_*, *d*_2_ is the estimated distance from *Sensor_C_* to *Beacon_L_*, and *d*_3_ is the estimated distance from *Sensor_R_* to *Sensor_C_*. We use the notation *d_k,n_* to represent the *k*-th estimated distance updated in the *n*-th round of iterations. From the true locations of three nodes, these three random variables have the parameters as *E*[*d_k,n_*] = 1, *V ar*(*d_k,n_*) = *σ*^2^, where *k* = 1, 2, 3.

According to our algorithm, since both *Beacon_L_* and *Sensor_R_* are neighbors to *Sensor_C_*, we get

(6) $$C_{n+1}=(1-2\delta )C_{n}+\delta R_{n}+\delta \left(d_{2,n}-d_{1,n}+L\right)$$

where *δ* is the step size constant specified in the algorithm. Similarly, *Sensor_R_* only has the neighbor *Sensor_C_*, and we have

(7) $$R_{n+1}=(1-\delta )R_{n}+\delta C_{n}+{\delta d}_{3,n}.$$

Formulas (6)–(7) can be viewed as a type of first order vector autoregression systems. The main purpose of this analysis is to show that the above system is stable. Precisely, we wish to show the variances of *C_n_* and *R_n_* after a long run remain finite. To solve (6)–(7) for *V ar*(*C_n_*) and *V ar*(*R_n_*), we first determine their covariance to be

$$\mathrm{cov}\left(C_{n},R_{n}\right)=\frac{(1-2\delta )\mathrm{Var}\left(C_{n}\right)+(1-\delta )\mathrm{Var}\left(R_{n}\right)}{3-3\delta }.$$

With the above result, using time series analysis, we further get

$$\begin{array}{l} \mathrm{Var}\left(C_{n}\right)=\frac{-\delta \left({3\delta }^{2}-13\delta +10\right)}{\delta^{3}-9\delta^{2}+22\delta -12}{\hat{\sigma }}^{2}\\ \mathrm{Var}\left(R_{n}\right)=\frac{-2\delta \left(3\delta^{2}-11\delta +7\right)}{\delta^{3}-9\delta^{2}+22\delta -12}{\hat{\sigma }}^{2}. \end{array}$$

From the last result, the variances of *C_n_* and *R_n_* are positive and remain finite when $\delta <3-\sqrt{5}\approx 0.764$. This concludes that the computed locations are wide sense stationary with a proper setting of the *δ* parameter. The *δ* parameter controls the step size in the algorithm, thus a large step size increases the fluctuation of *C_n_* and *R_n_* which in turns increases their variances.

From the above simple scenario, we see that while the measured signal strength fluctuates over the time, the estimated location also fluctuates and stays stationary around its true location, even for the sensor where none of its neighbors is a beacon. This stationary property suggests that cooperative approach for the spring-relaxation technique maintains stability in location estimation.

## 4. Simulation Results and Discussion

We use the following path loss model for the radio propagation [18].

(8) $${\text{log}}_{10}d=\frac{1}{10n}\left(P_{\mathrm{TX}}-P_{\mathrm{RX}}+G_{\mathrm{TX}}+G_{\mathrm{RX}}-X_{\alpha }+20\;{\text{log}}_{10}\lambda -20\;{\text{log}}_{10}(4\pi )\right)$$

where the involved variables are described in Table 1, and their default values follow the IEEE 802.15.4 standard for ZigBee [19].

### 4.1. Simulation for One-Hop Setup

In this section, we conduct simulation experiments to study the impact of protocol design parameters on the location estimation accuracy in one-hop setup using Matlab. The simulation environment is a coordinated square map of size 100 m × 100 m with *N_b_* = 5 beacons and *N_s_* = 50 sensors randomly deployed in uniform distribution. The five beacons are set into the four corners and the central point of the map.

#### 4.1.1. Parameter Design for Phase 1

In phase 1, the more iterations the algorithm executes, the closer the estimated location of a sensor approaches its stopping point. Due to the RSS ranging error, the eventual stopping point may not be the true location. The task of phase 1 is mainly to bring the estimated location to the stopping point, and then the location estimation continues with phase 2 to bring further from the stopping point to the true location. Therefore, for the performance of phase 1, our interest is to investigate the estimation error by measuring the distance between the estimated location of a particular sensor and its eventual stopping point rather than its true location. In other words, we measure

$$\mathrm{error}=\sqrt{{\left(x_{e,i}-x_{s,i}\right)}^{2}+{\left(y_{e,i}-y_{s,i}\right)}^{2}}$$

where (*x_e,i_*,*y_e,i_*) is the estimated location and (*x_s,i_*,*y_s,i_*) is the stopping location of *Sensor_i_*, *i* ∈ 𝕊.

**Figure 4.**
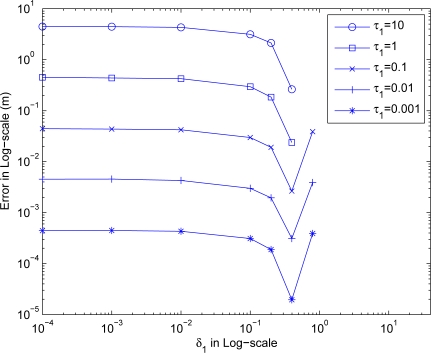
The estimation error with different *τ*_1_ and *δ*_1_.

Starting with some preparation experiments, we were able to narrow down the value range of *τ*_1_ and *δ*_1_. We then executed Algorithm 1 ten times for each pair of (*τ*_1_, *δ*_1_) combinations, and calculated the mean error and number of iterations by averaging over executions. In order to make the experiment more realistic, the initial guess of sensor locations are obtained randomly and independently for each piece of execution. Figure 4 shows the estimation error versus *δ*_1_ for different *τ*_1_ values.

As can be seen from the figure, *τ*_1_ value directly governs the estimation error where a smaller *τ*_1_ allows the algorithm to terminate and give a closer location to the stopping point. In other words, setting a large value for *τ*_1_ may give undesirable estimation location produced in phase 1. In the figure, using *τ*_1_ = 10 gives a 10 m error indicating that the produced location estimate is still 10 m away to the stopping point. Whereas using an very small *τ*_1_ = 0.001 gives a very small error of below 1 mm indicating that the produced location estimate almost reaches the stopping point. Notice that in the setup, the average distance between stopping points and corresponding actual positions of sensors is about 16.52 m. As a result, *τ*_1_ ≤ 0.1 looks appropriate by giving a mean error less than 0.1 m for all cases of *δ*_1_ values.

Moreover, the figure also shows that the estimation error generally reduces as *δ*_1_ increases for all the considered *τ*_1_ settings. For this setup, the estimation errors reach their lowest values at around *δ*_1_ = 0.4. The errors then jump to higher values as *δ*_1_ increases further indicating diverging of the algorithm. In terms of the accuracy, it is the best choice to fix *δ*_1_ = 0.4.

**Figure 5.**
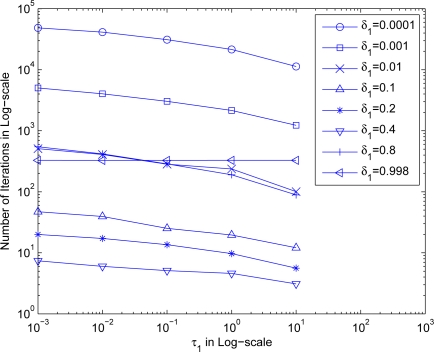
The convergence speed with different *τ*_1_ and *δ*_1_.

While the previous results suggest that *τ*_1_ ≤ 0.1 and *δ*_1_ = 0.4 gives a very close location to the stopping point, it is also necessary to consider the convergence speed measured by number of iterations in executions. Figure 5 plots the number of executed iterations using different combinations of *τ*_1_ and *δ*_1_. Since *τ*_1_ governs the termination of iterations, it can be expected that a smaller *τ*_1_ gives a more strict constraint on termination, thus lengthens the converging process and results in more number of iterations. Meanwhile, *δ*_1_ directly governs the convergence speed in the way that a larger *δ*_1_ gives a faster convergence. However, it is observed in Figure 5 that *δ*_1_ beyond 0.4 suddenly increases the number of iterations from tens to hundreds, suggesting that *δ*_1_ > 0.4 gives a too rapid position adjustment thus introduces a lot of oscillations in position updates. To give concurrent consideration to both the estimation error and the convergence speed, the optimal choice for phase 1 in this setup is *τ*_1_ = 0.001 and *δ*_1_ = 0.4.

#### 4.1.2. Parameter Design of Phase 2

Similar to that of phase 1, in Algorithm 2, the setting of *τ*_2_ and *δ*_2_ decides the termination condition of the loop in the algorithm, which in turns decides the location estimation error. In this phase, we investigate the evolution of location estimation error during the execution of Algorithm 2, where the location estimation error is measured by the distance between the estimated location and the true location of a particular sensor. Precisely,

$$\mathrm{error}=\sqrt{{\left(x_{e,i}-x_{i}\right)}^{2}+{\left(y_{e,i}-y_{i}\right)}^{2}}$$

where (*x_e,i_*,*y_e,i_*) is the estimated location and (*x_i_*,*y_i_*) is the true location of *Sensor_i_*, *i* ∈ 𝕊.

**Figure 6.**
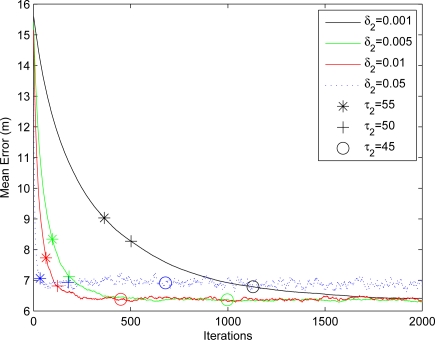
The evolution of location estimation error for the observed sensor with different *τ*_2_ and *δ*_2_.

The evolution of location estimation error for a particular sensor is plotted in Figure 6 where each curve is obtained by averaging over ten executions. In the figure, the symbols represent the termination point of the algorithm with various *τ*_2_ values. The figure shows three curves with different *δ*_2_ values: (i) when *δ*_2_ is set to a very small value, for example *δ*_2_ = 0.001, the algorithm takes more iterations to terminate, and the error drops smoothly over the iteration loops; (ii) when *δ*_2_ is set to a relatively large value, for example *δ*_2_ = 0.05, the algorithm generally takes much lower computation to terminate. Over the execution, the estimated location fluctuates greatly which indicates that the algorithm operates near the instability boundary due to a high value of *δ*_2_; (iii) when *δ*_2_ is set to a suitable value, for example *δ*_2_ = 0.01, the error reduces sharply in the beginning of the algorithm execution and then stays steadily with small fluctuations showing the stationary properties. This indicates that if the setting the chosen appropriately, the algorithm achieves quick convergence and stability with low error. To give concurrent consideration to both the estimation error and the convergence speed, the optimal choice for phase 2 in this setup is *τ*_2_ = 45 and *δ*_2_ = 0.01.

One extension of the algorithm is that we can let Algorithm 2 execute continuously by not applying *τ*_2_ for the termination condition. This allows the algorithm to cope with a certain mobility of sensors as this modification allows the sensors to continuously update and adjust their estimated location.

#### 4.1.3. Overall Accuracy

**Figure 7.**
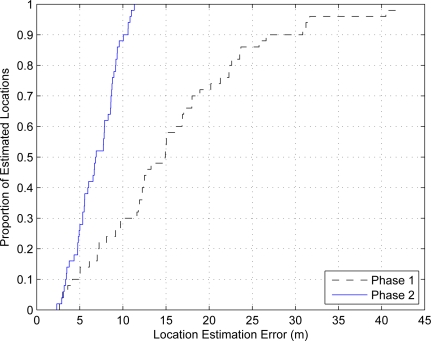
CDF of the location estimation error from two phases.

**Figure 8.**
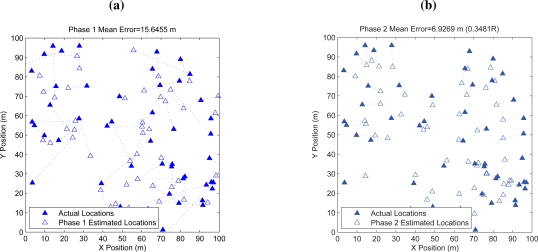
The estimated locations from two phases comparing to the true locations. (a) phase 1 estimated locations; (b) phase 2 estimated locations.

Using the chosen values for parameters, we test the overall accuracy of our proposed localization solution using the measure of location estimation error given in (9). Figure 8a depicts the estimated locations from phase 1 (shown in hollow triangles) and the corresponding true locations (shown in solid triangles). The errors are obvious. In Figure 8b, we show the estimated locations from phase 2 (shown in solid triangles) and the corresponding true locations (shown in hollow triangles). The refinement due to phase 2 is clearly illustrated with many close pairs of triangles and the matched shape of sensor deployment. Numerically, the average errors in phase 1 and 2 are 15.65 m and 6.93 m respectively. Normalizing to sensor transmission range *R*, phase 2 estimation gives 0.35*·R* in average. In Figure 7 which shows the CDF of the location estimation errors, we further see that with the effort of phase 2, 90% of the location estimation are improved from more than 30 m to 10 m.

#### 4.1.4. Performance Comparison to Related Approaches

To further show the performance advantages of our proposed method, we compare the accuracy performance of our proposed solution to other solution that can be deployed to perform localization in the same environment without additional specialized localization hardware. For this performance comparison, we select the *k*-nearest neighbors (kNN) approach, where *k* neighbors are the *k* pre-mapped geographic points whose RF fingerprints are closet matching to that of a sensor, and each of the k neighbors is weighed inversely proportional to its Euclidean distance in signal space from the sensor whose location is being estimated [23]. We implement a script following [23], and *k* = 3 is used in the script for this performance comparison.

One additional requirement for kNN approach is the database construction of RSS fingerprints through a site-survey prior to the localization. For the database construction, the more sampling points are performed, the better is the performance. With the same setup of the map size, the placement of beacons and the number of sensors, as well as the same path loss model and log-normal shadowing, we conduct performance studies for the kNN approach using different number of survey points via simulation, and report the results in Table 2. For each number of survey points, we execute kNN ten times and take average over ten times for the final mean estimation error. As can be seen, only for 25,600 (*i.e.*, 160 by 160) survey points, the kNN approach achieves just below 16 m, which is of 2.3 times the localization error of our proposed method. We also observe from the table that further increasing of the survey points to 250,000 does not have significant improvement in the accuracy. This confirms that our solution is a good RSS-based localization candidate.

**Figure 9.**
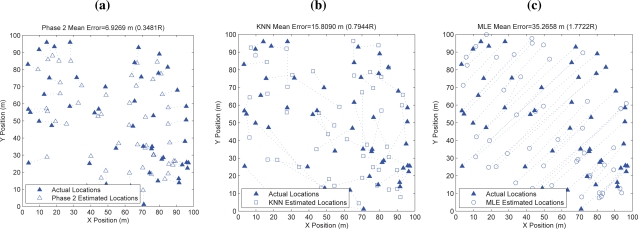
Location estimation produced by our algorithm versus KNN and MLE. (a) our estimated locations; (b) KNN estimated locations; (c) MLE estimated locations.

Furthermore, we compare our proposed solution to maximum-likelihood estimators (MLEs) for sensor location estimation [24] in the same one-hop setup. MLE works in a similar network setup that has a number of reference devices with known coordinates, and a number of blindfolded devices whose positions are to be estimated. With the combined range information between many pairs of devices and the known locations of reference devices, a Maximum-likelihood solution for the location of all of the blindfolded devices is determined. For this performance comparison, we used the MLE script implemented by Neal Patwari which can be obtained in [25]. Using the same system setup and sensor deployment shown in Figure 8, we execute ten trials of MLE, take mean over all ten sets of estimated locations produced in different trials as the resultant location estimates, and plot these location estimates in Figure 9c. The estimated locations produced by our algorithm as well as by kNN are also plotted for comparison. As can be seen in the figure, almost none of the location estimates can reach their actual locations, nor can them form a shape similar to that of the actual sensor deployment. Numerically, the mean localization error for MLE is 35.27 m, *i.e.*, 1.77*·R*, which is five times of our spring-relaxation solution.

### 4.2. Simulation for Two-hop Setup

We then enlarge the map to 350 m × 350 m to investigate the performance of our solution in two-hop networks. The same number of beacons and placement scheme are used for the beacons. Whereas the number of sensors *Ns* is gradually increased and sensors are randomly deployed which follows uniform distribution. Given the transmission range of beacon and sensor determined by propagation parameters listed in Table 1, the coverage of beacons is about 64.24%. In other words, about 64.24% sensors are of one-hop to some beacons, and about 35.76% sensors are of two-hop cases. The network connectivity (*i.e.*, average number of neighbors) can be calculated basing on a specific network topology. After applying our localization algorithm, the percentage of unsolved sensor nodes (*i.e.*, the percentage of sensors whose locations cannot be estimated using the localization technique) is computed, and the mean localization error of the location estimation is compared with that using MLE in the same network settings, as listed in Table 3.

Given the same beacon coverage, it can be expected that as the number of sensors increases, the network connectivity will increase, thus more sensors’ locations can be resolved using spring-relaxation, which is reflected in Table 3. It can also be observed that the average estimation error of our algorithm decreases when more neighbors present in the cooperative localization, whereas the average estimation error of MLE generally maintains at 170 m. The simulation results suggest that our algorithm outperforms MLE in terms of accuracy in the same network setup.

## 5. Conclusions

In this paper, we proposed a localization solution consisting of two phases of localization algorithm based on spring-relaxation technique for large-scale distributed wireless sensor networks. We showed that spring-relaxation technique is suitable for multi-hop cooperative localization. Our proposed solution is based on the spring-relaxation technique and thus inherits its implementation simplicity. Moreover, our design requires only a few beacons with known locations to compute the location estimates of all sensors. In our simulation experiments, we demonstrated the overall accuracy of our design and favorable performance over both kNN and MLE approaches.

## Figures and Tables

**Table 1.** The variables involved in Equation (8).

Variable	Definition
*d*	estimated distance between the transmitter and the receiver [m]
*P_TX_*	transmitted power level [dBm]
*P_RX_*	received power level [dBm]
*G_TX_*	antenna gain of the transmitter [dBi]
*G_RX_*	antenna gain of the receiver [dBi]
λ	signal wavelength [m]
*n*	path loss exponent
*X_α_*	Gaussian random variable with a standard deviation of *α*

**Table 2.** The accuracy performance of the kNN approach with various number of sampling points equally spread on a 100 m by 100 m map.

The number of survey points	Average accuracy (m)
5 by 5	24.88
10 by 10	22.15
20 by 20	18.90
40 by 40	17.73
80 by 80	16.11
160 by 160	15.81
500 by 500	15.31

**Table 3.** Sensor deployment parameters and accuracy performance of the proposed algorithm versus MLE on a 350 m by 350 m square map.

Ns	Connectivity	Unsolved (%)	Mean error (m)	Mean error of MLE (m)
100	4.13	13%	42.4	172.72
200	5.56	5%	26.02	176.93
300	6.06	2.33%	24.72	160.86
400	7.03	0.6%	23.94	171.24
500	7.99	0.5%	23.12	174.31
600	8.82	0.33%	22.63	161.91
